# *In vitro* and *in vivo* study of the inhibition of ubiquinone biosynthesis in *Leishmania (Leishmania) amazonensis* using the combination of buparvaquone and 4-nitrobenzoate as a strategy for the discovery of new therapeutic targets

**DOI:** 10.1128/aac.00824-25

**Published:** 2025-10-07

**Authors:** Juliana Tonini Mesquita, Gabriel Cândido Moura, Tainá Cavalcante, Jenicer Kazumi Umada Yokoyama Yasunaka, Manoel Aparecido Peres, Claudia Blanes Angeli, Noemi Nosomi Taniwaki, Beatriz Simonsen Stolf, Giuseppe Palmisano, Alejandro Miguel Katzin

**Affiliations:** 1 Department of Parasitology, Institute of Biomedical Sciences, University of São Paulo 28133, São Paulo, Brazil; 2 Electron Microscopy Nucleus, Institute Adolfo Lutz 89119, São Paulo, São Paulo, Brazil; The Children's Hospital of Philadelphia, Philadelphia, Pennsylvania, USA

**Keywords:** leishmaniasis, *Leishmania (Leishmania) amazonensis*, treatment, ubiquinone therapeutic target, combination therapy

## Abstract

Leishmaniasis is a parasitic disease caused by several species of the genus *Leishmania,* considered one of the most dangerous neglected tropical diseases in the world. Treatment options are limited and toxicity exists, requiring an urgent search for new therapies. *Leishmania* differs from mammalian cells by having unique mitochondria, which have been considered an important therapeutic target. Ubiquinone is found in the inner membrane of mitochondria and plays a critical role in the electron transport chain, which is essential for cell viability. The combination of buparvaquone and 4-nitrobenzoate seems an interesting option due to their action on these targets. The aim of the present work was to investigate the effects of this combination against *Leishmania (Leishmania) amazonensis* infections *in vitro* and *in vivo* and to elucidate the possible mechanism of action of 4-nitrobenzoate. The results showed that the drug combination tested in promastigotes has an additive or indifferent effect. In the intracellular amastigotes, when buparvaquone was tested with fixed concentrations of 4-nitrobenzoate, concentrations that had no effect alone potentiated the effect of buparvaquone by two to four times. The mechanism of action of 4-nitrobenzoate in promastigote forms suggests a reduction in ubiquinone 6 levels, an alteration in mitochondrial membrane potential, and an increase in reactive oxygen species. *In vivo* treatment with a topical formulation (buparvaquone 0.1% and 4-nitrobenzoate 0.1%) in infected mice showed a reduction in parasite burden of 57% compared to the control group, suggesting that this combination may represent a promising therapeutic strategy for future treatment of this disease.

## INTRODUCTION

Leishmaniasis is a parasitic disease endemic in 99 countries, considered one of the most dangerous neglected tropical diseases, second only to malaria in causes of death (1, 2). Leishmaniasis is caused by species of *Leishmania* and has a broad spectrum of clinical manifestations, grouped into cutaneous, mucocutaneous, and visceral presentations (1, 3). The cutaneous form is the most common, often resulting in lifelong scars, disability, or stigma, whereas the visceral form, the most severe, can be fatal in over 95% of cases if untreated (4, 5).

The treatment of leishmaniasis depends on many factors, including the type of disease, concomitant pathologies, parasite species, and geographic location (5). In the absence of a vaccine, the treatment of human leishmaniasis depends on chemotherapy (6). The drugs used for treatment are pentavalent antimonial, amphotericin B, miltefosine, pentamidine, and paromomycin (4). The available chemotherapeutic drugs have problems related to efficacy, treatment duration, emergence of parasite resistance, toxicity, and high cost, which justifies the search for new drugs (4, 7). These limitations highlight the need for new pharmaceutical prototypes that prioritize effective therapeutic targeting in *Leishmania* while ensuring safety for humans. In addition, the chosen therapy must be easier to administer through a skin lesion (topical formulation), resulting in better patient adherence to treatment.

Mitochondria are crucial for parasite survival and have been widely explored as targets for antiparasitic drugs (8). Currently available studies on trypanosomatid mitochondria are scarce. The available evidence supports the essential role of this organelle and its peculiarities compared to its human counterpart, making it an attractive and safe candidate for drug development (8). Ubiquinone (UQ) is widely distributed in biological systems and carries out the well-characterized role as a redox component involved in the transfer of electrons and protons in mitochondria (9). Isoprenoid-derived compounds, including dolichols or UQ, have been shown to be important for cell viability and proliferation in different trypanosomatid species (10).

Buparvaquone (BPQ), a drug used to treat theileriosis, has shown promising anti-*Leishmania* activity (11). BPQ acts selectively against the parasite’s electron transport system at UQ, resulting in competitive inhibition of the parasite’s electron transport respiratory chain (12). Another drug evaluated in this work was 4-nitrobenzoate (4-NB), which inhibits the biosynthesis of 4-hydroxybenzoate, thereby reducing UQ in mammalian cells (13). Based on this, our study aimed to investigate the potential of the UQ biosynthesis pathway as a drug target in *Leishmania* by evaluating the combined effect of BPQ and 4-NB in both *in vitro* and *in vivo* models of cutaneous leishmaniasis (CL). This strategy may contribute to the development of more effective and targeted treatments with fewer side effects and improved patient compliance.

## RESULTS

### *In vitro* anti-*Leishmania* activity, mammalian cytotoxicity, and selectivity index of BPQ and 4-NB

The *in vitro* effects of BPQ and 4-NB were studied in extracellular and intracellular forms of *Leishmania (Leishmania) amazonensis* for 72 hours. BPQ was active against both forms of *Leishmania*, with an effective concentration 50% (EC_50_) of 0.01 and 1.45 µM against promastigotes and intracellular amastigotes, respectively. 4-NB was active only against promastigote forms, with an EC_50_ of 1.94 mM. Amphotericin B and miltefosine were included as positive control drugs against leishmaniasis, with EC_50_ values of 0.58 and 29.87 µM against promastigote forms and 0.41 and 8.52 µM against intracellular amastigote forms, respectively. The cytotoxicity was assayed in the human THP-1 cell line differentiated into macrophage-like cells, showing that BPQ and 4-NB had cytotoxic concentrations of 50% (CC_50_) of 132.47 µM and 13.36 mM, respectively. The selectivity index (SI) for BPQ was 91.36, while for amphotericin B and miltefosine, it was 805.20 and 10.27, respectively (Table 1).

**TABLE 1:** *In vitro* anti-*Leishmania* activity, cytotoxicity against mammalian cells, and selectivity index (SI) of buparvaquone, 4-nitrobenzoate, amphotericin B, and miltefosine^*a*^

Drugs	Promastigotes EC_50_ (µM) (±SD)	Amastigotes EC_50_ (µM) (±SD)	Cytotoxicity CC_50_ (µM) (±SD)	SI
Buparvaquone	0.01 ± 0.00	1.45 ± 0.53	132.47 ± 38.40	91.36
4-Nitrobenzoate^*b*^	1.94^*b*^ ± 0.29	NA^*b*^	13.36^*b*^ ± 2.85	ND
Amphotericin B^*c*^	0.58 ± 0.15	0.41 ± 0.17	330.13 ± 95.25	805.20
Miltefosine^*c*^	29.87 ± 4.42	8.52 ± 1.02	87.47 ± 3.68	10.27

^*a*^ EC_50_, effective concentration 50%; CC_50_, cytotoxic concentration 50% using THP-1 cells differentiated into macrophage-like cells; SI, selectivity index (the ratio between CC_50 _and EC_50 _against amastigotes); ±SD, standard deviation; NA, not active at the concentrations tested; ND, not determined.

^*b*^ Values expressed in millimolar (mM).

^*c*^ Standard drug (drugs for clinical use). Values represent average and standard deviations of three independent experiments.

### *In vitro* combination therapy with BPQ and 4-NB against *Leishmania (Leishmania) amazonensis*

**Fig 1:**
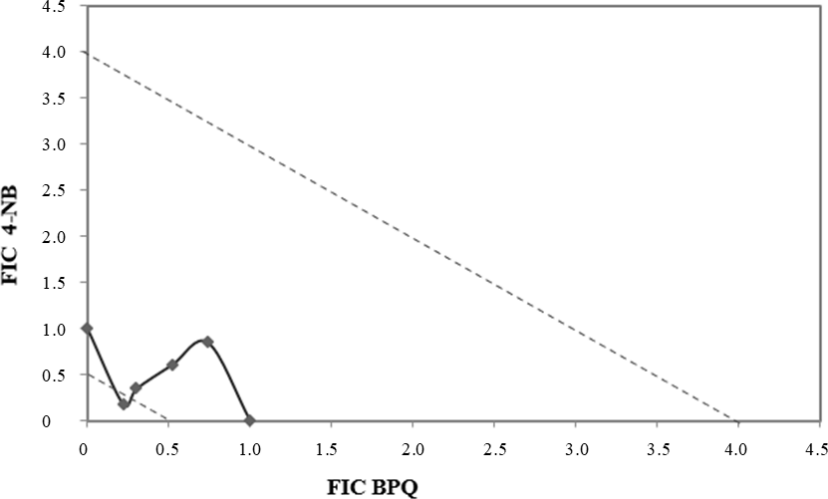
Isobologram based on the FIC values of the combination between BPQ and 4-NB against promastigotes. The points correspond to the FIC values of the combinations, which were classified as indifferent interactions. The points corresponding to the FIC of the associations were connected by a trend line that was in the range between 0.5 and 4, identified by the dotted lines. Values represent an average of three independent experiments.

The therapeutic combination of BPQ and 4-NB was tested against promastigotes for 72 hours. The modified isobologram method was used, and the dose-response curves were calculated individually and in combination using proportions 5:0; 4:1; 3:2; 2:3; 1:4; and 0:5. To construct the isobologram and classify the nature of the interaction, the fractional inhibitory concentrations (FICs) were calculated for each combination tested, and the sum of FIC (∑FIC) showed 1.59, 1.13, 0.65, and 0.40. The mean of ∑FIC was 0.94, indicating that the interaction was additive or indifferent (Fig. 1).

Drug potentiation between BPQ and 4-NB was tested because only BPQ showed activity against intracellular amastigotes in macrophage-like cells treated for 72 hours. Since 4-NB did not show activity in amastigotes under the conditions tested (Table 1), we performed a potentiation analysis of BPQ using fixed doses of 4-NB. The results showed that BPQ alone had an EC_50_ of 1.53, and when 4-NB was added at concentrations of 2.0, 1.0, 0.5, and 0.25 mM, the EC_50_ varied from 0.73 to 0.36, showing that 4-NB potentiated the effect of BPQ from 2.10 to 4.25 times (Table 2).

**TABLE 2:** *In vitro* potentiation of drugs between BPQ and 4-NB against macrophage-like cells infected with intracellular amastigotes of *Leishmania (Leishmania) amazonensis^a^^,b^*

Drugs	EC_50_ (µM) (±SD)	Potentiation
BPQ µM (alone)	1.53 ± 0.45	ND
BPQ µM + 4-NB 2.00 mM	0.36 ± 0.25	4.25
BPQ µM + 4-NB 1.00 mM	0.40 ± 0.24	3.83
BPQ µM + 4-NB 0.50 mM	0.63 ± 0.33	2.43
BPQ µM + 4-NB 0.25 mM	0.73 ± 0.46	2.10

^*a*^ EC_50_, effective concentration 50%; potentiation: the ratio of BPQ alone and BPQ with 4-NB; and ±SD, standard deviation; ND, not determined.

^*b*^ Values represent an average and standard deviation of three independent experiments.

### *In vitro* study of the mechanism of action of 4-NB against *Leishmania (Leishmania) amazonensis*

**Fig 2:**
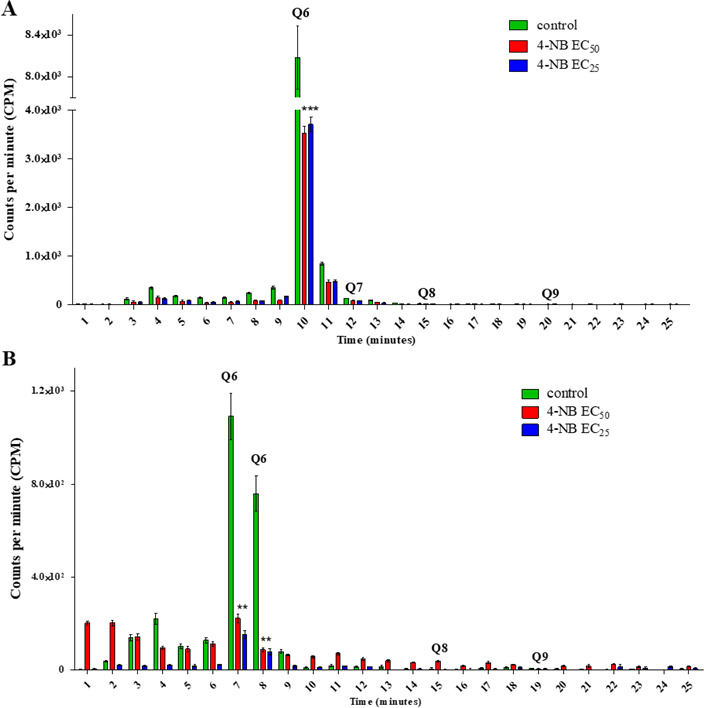
UQ abundance estimated by RP-HPLC after treatment of promastigotes with 4-NB. UQ was identified through separate metabolic labeling by RP-HPLC using the radioactive precursor 4-hydroxybenzoic acid [ring-^14^C] (0.5 µci/mol) (**A**) and mevalonic acid, ammonium salt R-[5-^3^H] (2 µci/mol) (**B**) in promastigote forms untreated (control) and treated with 4-NB at concentrations of EC_50_ and EC_25_ for 72 hours. Commercial standards UQ 6, UQ 7, UQ 8, and UQ 9 were used as controls. Values represent averages and standard deviations from a representative assay of two independent experiments.

4-NB potentiated the effect of BPQ against amastigote forms of *Leishmania (Leishmania) amazonensis*. Since the mechanism of action of BPQ against *Leishmania* is already known, we decided to investigate the mechanism of action of 4-NB in promastigote forms of *Leishmania (Leishmania) amazonensis* (unique form that 4-NB presents activity alone), based on the hypothesis that 4-NB could inhibit UQ synthesis. To this end, promastigote forms treated with 4-NB for 72 hours, as well as untreated controls, were labeled with the markers 4-hydroxybenzoic acid [ring-^14^C] (0.5 µci/mol) (Fig. 2A) or mevalonic acid and ammonium salt R-[5-^3^H] (2.0 µci/mol) (Fig. 2B). Figure 2A shows the peaks corresponding to the chromatographic elution profile of the UQ 6 commercial standard, observed in fraction 10. A quantitative analysis showed that samples treated with 4-NB had a significant reduction of 56.89% (treated with EC_50_) and 54.66% (treated with EC_25_) in counts per minute (CPM) compared to control (untreated parasites) (Fig. 2A). Using mevalonic acid, ammonium salt R-[5-^3^H] precursor for radioactive labeling of 4-NB, treated and untreated samples presented peaks corresponding to the elution time of the UQ 6 standard, observed at fractions 7 and 8. At fraction 7, the CPM peaks showed reductions of 79.49% (treated with EC_50_) and 85.85% (treated with EC_25_) compared to the control. Similar reductions were observed at time 8, when the CPM peaks showed 88.42% (treated with EC_50_) and 89.41% (treated with EC_25_) decrease compared to the control (Fig. 2B). These results suggest that the mechanism of action of 4-NB in *Leishmania (Leishmania) amazonensis* promastigote forms is related to the biosynthesis of ubiquinone-6. Other commercial standards were used as controls in addition to UQ 6, such as UQ 7, UQ 8, and UQ 9, but low values of scintillation CPM were observed.

**Fig 3:**
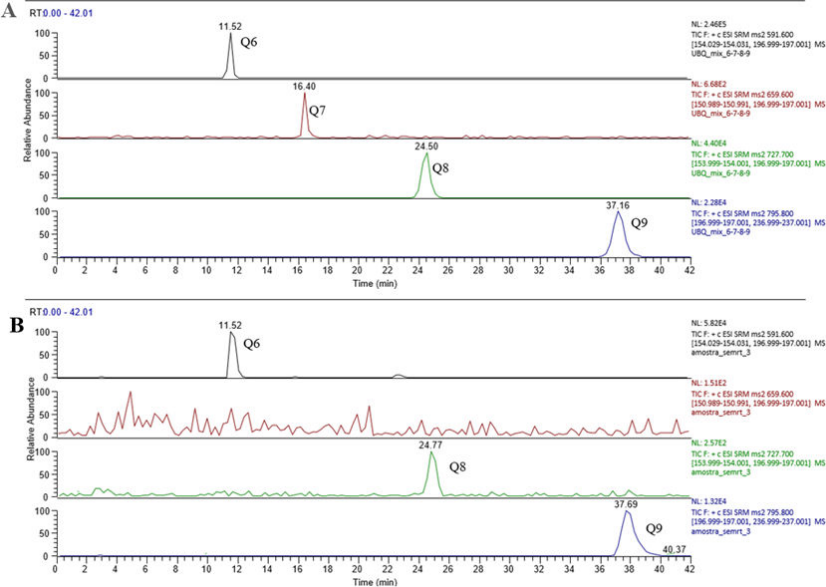
LC-MS/MS analysis of UQ in promastigote forms. Extracted ion chromatograms of transition precursor ion > product ions for each UQ. Q6: 591 *m/z* > 197 and 154 *m/z*; Q7: 659 *m/z* > 197 and 151 *m/z*; Q8: 727 *m/z* > 197 and 154 *m/z*; and Q9: 795 *m/z* > 197 and 237 *m/z*. Detection of UQ 6, UQ 7, UQ 8, and UQ 9 commercial standards (**A**). Detection of UQ6, UQ 8, and UQ 9 in *Leishmania (Leishmania) amazonensis* promastigotes extract (**B**).

Liquid chromatography-mass spectrometry (LC-MS) in positive ionization mode was used to identify UQ in promastigotes. Commercial standards UQ 6, UQ 7, UQ 8, and UQ 9 were used as controls. Figure 3A shows the peaks for the commercial standards at 11.52 minutes (UQ 6), 16.40 minutes (UQ 7), 24.50 minutes (UQ 8), and 37.16 minutes (UQ 9). Figure 3B shows that *Leishmania (Leishmania) amazonensis* promastigotes extract had the same adduct pattern and time as UQ 6, UQ 8, and UQ 9, confirming the presence of these molecules in the promastigote forms of *Leishmania (Leishmania) amazonensis*.

**Fig 4:**
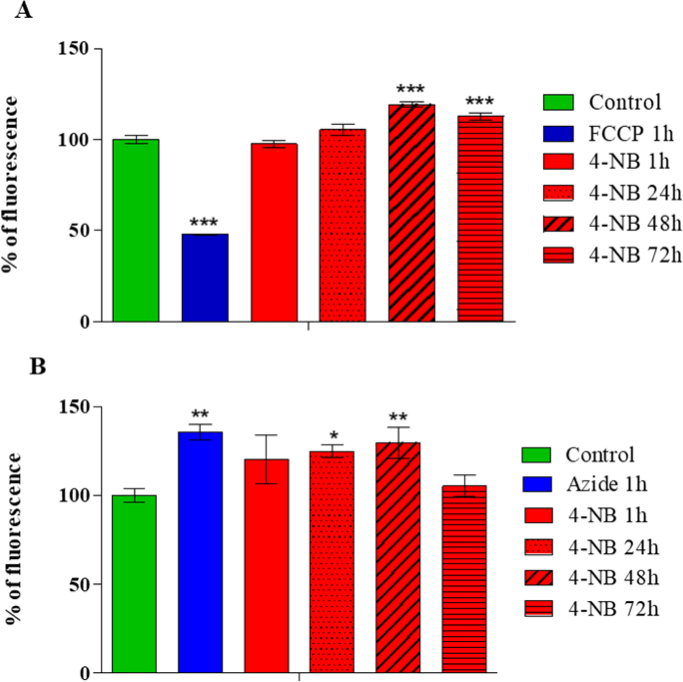
Evaluation of mitochondrial membrane potential using the JC-1 fluorescent probe (**A**) and ROS production using the CM-H_2_DCF-DA probe (**B**). Promastigote forms were treated with EC_50_ of 4-NB for 1, 24, 48, and 72 hours. Controls correspond to untreated promastigote forms and promastigotes treated with FCCP (positive control) (**A**). Controls correspond to untreated promastigote forms and those treated with azide (positive control) (**B**). The fluorescence of the parasites was normalized to untreated promastigote forms (control), represented as 100% (**A and B**). Values represent averages and standard deviations from three independent experiments.

The mechanism of action of 4-NB was also analyzed by evaluating mitochondrial membrane potential and reactive oxygen species (ROS) production in promastigote forms. Figure 4A shows that promastigote forms treated with 4-NB exhibited fluorescence levels similar to those of the untreated control at 1 and 24 hours and displayed significant increases of 20% and 13% of fluorescence at 48 and 72 hours, respectively, indicating the hyperpolarization of mitochondrial membrane potential (Fig. 4A). Promastigote forms treated with carbonyl cyanide *p*-trifluoromethoxyphenylhydrazone (FCCP) for 1 hour were used as a positive control for depolarization of the mitochondrial membrane (Fig. 4A). The results in Fig. 4B show ROS production in treated and untreated promastigotes. Untreated promastigotes were considered basal ROS control, while promastigotes treated with sodium azide for 1 hour were used as a positive control for increased ROS production. After 1 and 72 hours of treatment with 4-NB, the fluorescence was similar to the control group (untreated), while promastigote forms treated for 24 and 48 hours showed 25%–30% increases in fluorescence, respectively, indicating a significant increase in ROS.

**Fig 5:**
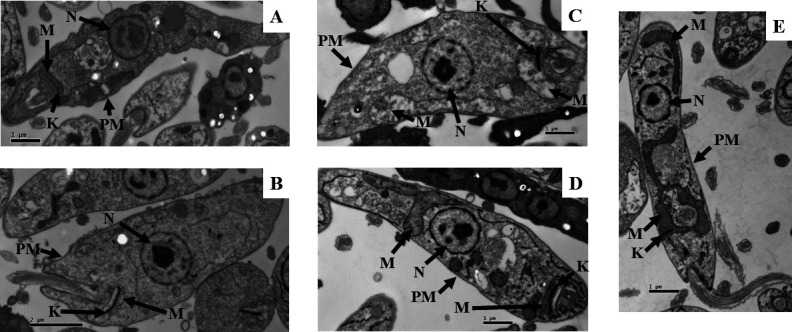
Ultrastructural analysis of promastigote forms, untreated and treated with 4-NB. Promastigotes were treated with an EC_50_ concentration of 4-NB for 1 (**A**), 24 (**B**), 48 (**C**), and 72 hours (**D**) and untreated promastigote forms (control) (**E**). Images acquired by transmission electron microscopy. (** A, C**, **D,** and **E**) 1 µm bar; (**B**) 2 µm bar. The arrows indicate: mitochondria (M), kinetoplast (K), nucleus (N), and plasma membrane (PM). Magnification: 15,000 times.

We also analyzed the effects of 4-NB on the ultrastructure of promastigotes. Parasites were treated with EC_50_ of 4-NB for 1 hour (Fig. 5A), 24 hours (Fig. 5B), 48 hours (Fig. 5C), and 72 hours (Fig. 5D), and untreated promastigote forms were used as a control for the experiment (Fig. 5E). The untreated parasites displayed a preserved nucleus (N), plasma membrane (PM), mitochondria (M), and kinetoplast (K), a dense region of mitochondrial DNA located near the flagellar pocket (Fig. 5E). Parasites treated with 4-NB for 1 and 24 hours (Fig. 5A and B) appeared to have their organelles as preserved as the control group (Fig. 5E). However, after 48 hours of treatment (Fig. 5C), the parasite showed swollen kinetoplast and mitochondria, a breakdown of mitochondria cristae and nuclear membrane with irregular heterochromatin. After 72 hours of treatment with 4-NB, the nucleus and plasma membrane were preserved, while the mitochondria and kinetoplast were enlarged, and the mitochondrial cristae were altered (Fig. 5D).

### *In vivo* activities of the combination of BPQ with 4-NB against *Leishmania (Leishmania) amazonensis*

**Fig 6:**
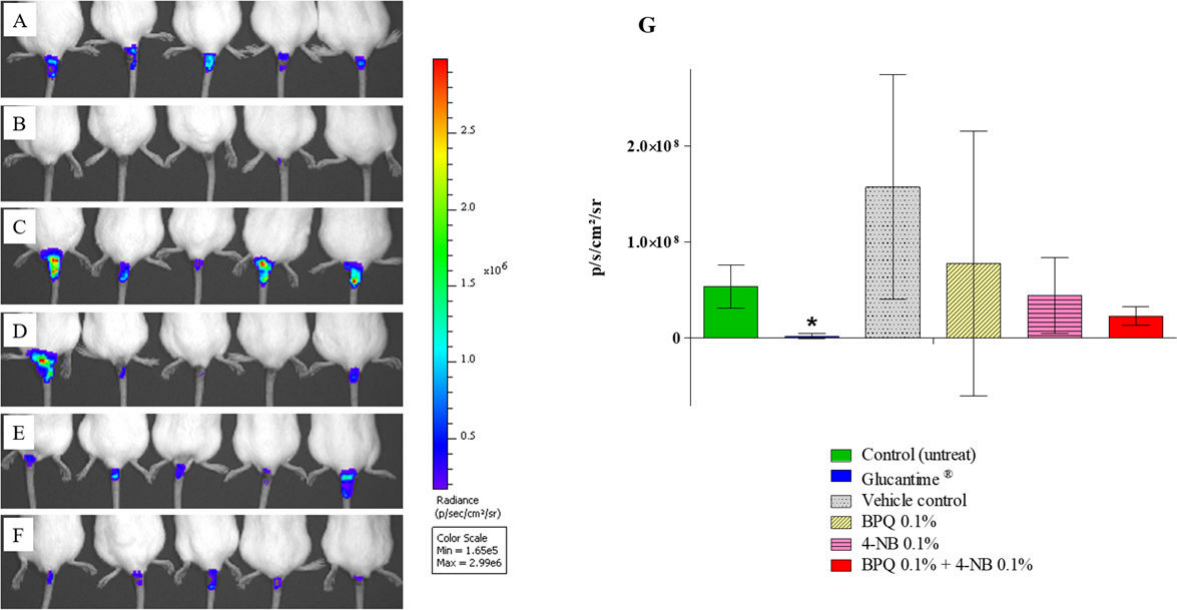
Bioluminescence imaging of mice infected with *Leishmania (Leishmania) amazonensis*. Six different groups were used: (**A**) control group (untreated); (**B**) group treated with standard drug Glucantime (intralesional); (**C**) vehicle control group (cream without active ingredient); (**D**) BPQ 0.1% drug control group; (**E**) 4-NB 0.1% drug control group; and (**F**) group combination of BPQ 0.1% + 4 NB 0.1%. (**G**) Estimation of parasite loads by bioluminescence in the six groups: control (untreated); standard drug Glucantime (intralesional); vehicle control (topical); BPQ 0.1% (topical); 4-NB 0.1% (topical); and combination of BPQ 0.1% and 4-NB 0.1% (topical) (**G**). The bars indicate the averages and standard deviations of the bioluminescence of the lesions in each group (five animals per group) from a representative experiment of two independent experiments. p/s/cm^2^/sr, photons/second/square centimeter/steradian.

The *in vitro* potentiating effect of the BPQ with 4-NB in intracellular amastigote forms seems promising and was therefore evaluated *in vivo* studies. The topical route of administration was chosen for this study. In the *in vivo* combination experiment, mice infected with *Leishmania (Leishmania) amazonensis* at the base of the tail were used to test the combination of the drug BPQ 0.1% with 4-NB 0.1%. Six distinct groups were used in this experiment (five mice per group): A—control group (untreated) (Fig. 6A); B—group treated with the clinical standard drug Glucantime intralesional (Fig. 6B); C—vehicle control group (formulation without active ingredient) (Fig. 6C); D—BPQ 0.1% drug control group (Fig. 6D); E—4-NB 0.1% drug control group (Fig. 6E); and F—combination of BPQ 0.1% with 4-NB 0.1% (Fig. 6F). Groups C, D, E, and F were treated topically for 28 consecutive days, Group B was treated for 14 days. The parasite load was determined by bioluminescence imaging of the lesion at the end of treatment, as shown in Fig. 6. The bioimaging result showed a 57% reduction in parasite loads in the group treated with the combination of BPQ 0.1% and 4-NB 0.1% compared to the control (untreated) group (Fig. 6G). The group treated with the standard drug Glucantime significantly reduced the parasite load by 95% (Fig. 6G). The other control groups used in the experiment, the vehicle control, BPQ 0.1% drug control, and 4-NB 0.1% drug control, showed no statistical differences in parasite load compared to the untreated control, besides showing a reduction of 17% in the control treated only with 4-NB.

## DISCUSSION

*Leishmania* is a protozoan parasite of the family Trypanosomatidae that is responsible for diseases known as leishmaniasis (6). This study focuses on *Leishmania (Leishmania) amazonensis*, the causative agent of CL and diffuse CL (14). Treatment of leishmaniasis is limited to a few drugs, such as pentavalent antimonials, which have remained the first choice for several decades in many endemic areas, including Brazil, despite their low efficacy rates (14). The use of intralesional therapy with antimonials was introduced with the aim of reducing the adverse effects compared to their systemic administration, where the recommended dose is 1–5 mL (applied in five sites per lesion) every 3–7 days until healing, and the reported adverse events include local irritation, edema, erythema, pruritus, and pain (3). Miltefosine is an oral drug approved by the Brazilian health system as an alternative for the treatment of CL, with cure rates of 70% (14, 15), but its use is limited to some species of *Leishmania* (16). Other drugs may be used when the first-line drug fails, such as amphotericin B and pentamidine (via the parenteral route), which also have issues in terms of toxicity (14, 16). As is evident, there is an urgent need for new therapeutic alternatives.

The identification of appropriate pharmacological targets in conjunction with biological pathways constitutes the initial stage of drug discovery (17). Available studies on trypanosomatid mitochondria have indicated an essential role for this organelle in the search for new candidates for drug development (8). UQ is a redox-active lipid present in all cellular membranes, and its functions are known to act as a mobile electron carrier in the mitochondrial respiratory chain (18). Therefore, inhibitors of this pathway were explored as potential targets for the development of new drugs. We focused on the inhibition of UQ biosynthesis in *Leishmania (Leishmania) amazonensis* using BPQ combined with 4-NB *in vitro* and *in vivo* infection models.

The combination drug regimen often leads to greater therapeutic efficacy than monotherapy, and other benefits may include less toxicity, delay or prevention of drug resistance development, and other favorable effects (19). However, to evaluate combination therapy properly, it is necessary to first understand the activity of each drug individually.

A series of hydroxynaphthoquinones were synthesized in the 1980s, and this class of drugs demonstrates activity against protozoa (11, 20). BPQ, a hydroxynaphthoquinone, acts selectively against the parasite’s electron transport system at UQ-cytochrome bc1 complex, leading to competitive inhibition of the parasite’s electron transport respiratory chain but not the vertebrate host’s at the parasite inhibitory levels (12). Cytochrome bc1 is thus a promising target for novel anti-*Leishmania* drugs, and further improvements on the BPQ scaffold are warranted for the development of enhanced therapeutics (21). For this reason, 4-NB was chosen as another inhibitor to be combined with BPQ. The literature indicates that 4-NB inhibits 4-hydroxybenzoate and dose-dependently decreases UQ in mammalian cells (13).

*Leishmania* parasites exhibit two principal life stages: the extracellular promastigotes, which are present in the sandfly (vector), and the intracellular amastigotes, present in mammalian cells (host). The activity against these two forms was investigated, and the results show that BPQ is active against both, while 4-NB showed activity only against promastigote forms. The literature describes the activity of BPQ in many species of *Leishmania* (11, 22–24), and our results corroborate these data. No data were found in the literature describing the activity of 4-NB against *Leishmania* promastigote forms. One study describes activity against *Plasmodium falciparum*, using concentrations of 4-NB in the millimolar range (25), similar to the data found in our study. Another important parameter considered in this study was the safety of the drugs in mammalian cells. The cytotoxicity of BPQ and 4-NB was tested and showed a higher CC_50_ than the standard drug miltefosine. In addition, the selectivity of BPQ was nine times higher than that of miltefosine.

The study of combination using the modified isobologram method with BPQ and 4-NB in promastigote forms showed an additive or indifferent interaction according to the classification by ODDS (26). Since in amastigote forms only BPQ showed activity, the combination included 4-NB at fixed doses, in which it did not show activity alone. The results confirmed the potentiation of BPQ by 4-NB, with EC_50_ values reduced by two to four times in a dose-dependent manner. Verdaguer et al. (25) showed similar results with 4-NB in *Plasmodium falciparum,* in which 4-NB potentiated the effect of atovaquone, an analog of BPQ (25). *Leishmania*, as an obligate intracellular parasite, is shielded from conventional chemotherapy, which does not readily diffuse through the host cellular membrane (20). BPQ, due to its lipophilic nature (27), when used together with 4-NB, may facilitate the entry of 4-NB into infected macrophages, thereby increasing or potentiating its leishmanicidal efficacy. The promising data found encouraged our group to search for evidence of the mechanism of action of 4-NB in *Leishmania (Leishmania) amazonensis*.

UQ is a molecule composed of a benzoquinone ring and an isoprene side chain, which varies between 6 and 10 isoprene units in different organisms and serves as a membrane anchor (28, 29). As previously described, 4-NB inhibited 4-hydroxybenzoate: polyprenyl transferase (UQ2) in a competitive manner and dose-dependently decreased UQ levels in mammalian cells without accumulation of UQ intermediates (13). To check whether this also occurred in *Leishmania*, UQ levels were analyzed using two different metabolic markers that are used as UQ precursors. With both radioactive precursors, there was a reduction in the peak related to UQ 6 in the group treated with 4-NB in a dose-dependent manner. We then employed liquid chromatography coupled to mass spectrometry to show that *Leishmania (Leishmania) amazonensis* promastigote forms exhibited the same adduct pattern and retention times as the UQ 6, UQ 8, and UQ 9 standards, confirming the presence of these molecules in the parasite.

In the literature, Ellis et al. (9) also showed that UQ 7, UQ 8, and UQ 9 are present in *Leishmania (Leishmania) amazonensis* promastigote forms, while UQ 9 was the only homologous UQ detected in the intracellular amastigote forms (9). Our results indicate the presence of UQ 6, UQ 8, and UQ 9 in the promastigote forms, confirming UQ 8 and UQ 9 but diverging with respect to UQ 6. Ellis et al. (9) did not describe the origin of the *Leishmania* strain used in their publication, which may also explain the difference found in our studies. Other hypotheses for this difference could be variations in incubation time, temperature, and other cultivation conditions that may influence the parasite respiration, affecting the formation of the isoprene side chain, for example, leading to the production of UQ 6 instead of UQ 7. Another difference in our experiment was the use of culture Schneider’s medium, while Ellis et al. (9) used M199 medium, which contains menadione, a synthetic naphthoquinone that can be converted into menaquinone. Macedo et al. (30) showed that *Plasmodium falciparum* is able to synthesize different homologs of UQ, depending on the given intermediate (30). Our results also showed a reduction in the concentration of UQ in promastigote forms of *Leishmania (Leishmania) amazonensis* treated with 4-NB, corroborating the data from Forsman et al. (13) in mammalian cells (13).

Investigations into the mechanism of action of 4-NB were carried out using fluorescent probes and ultrastructural analyses. Our results showed that the mitochondrial membrane potential was increased (after 48 and 72 hours), while the production of reactive species was increased (after 24 and 48 hours) in a time and dose-dependent manner. In addition, morphological analysis showed that mitochondria were swollen, and the cristae were broken down (after 48 hours), altered, and enlarged (after 72 hours), in agreement with the other results. UQ is crucial for the electron transport chain, as it transfers electrons from complexes I and II to complex III (31). It is also essential for mitochondrial ATP production and plays a significant role in the generation of mitochondrial ROS (31–33), which is consistent with our results.

Variations in mitochondrial membrane potential can be a consequence of various events but are essential to maintain the physiological function of the ATP-generating respiratory chain, and in its absence, cells can cease ATP synthesis, leading to cell death (34, 35). ROS can also be generated in the mitochondria during the oxidative phosphorylation process, although there are fluctuations in the basal levels, which can be crucial for the physiology of the cell (36). Oxidative stress increases the concentration of ROS, and their removal prevents toxicity (36). UQ functions in the mitochondrial respiratory chain and serves as a lipophilic antioxidant (37), therefore, its inhibition with 4-NB justifies the damages described.

Pathological disorders can impair the skin barrier, and CL is an infection of the dermal layers of the skin (38). Croft et al. (22) describe the excellent *in vitro* activity of BPQ against *Leishmania (Leishmania) donovani* amastigote forms, but its efficacy *in vivo* was limited by the poor bioavailability due to its very low aqueous solubility (22, 39). Other studies described in the literature have used BPQ, but in most cases, the formulation was improved using nanotechnologies (24, 40, 41). Although most treatments are delivered by other routes, topical drugs can effectively reach infected tissues in simple cases (38). The most promising finding of this study was the potentiation of BPQ by 4-NB in the amastigote forms, which are the most relevant forms for drug discovery, prompted the continuation of the work in the *in vivo* model. To study the combination of BPQ with 4-NB in this model, a topical route of administration was chosen because of its advantages over the intralesional use of Glucantime in the clinic.

Topical application is advantageous as it allows local and targeted drug exposure, thereby reducing the potential side effects and the need to carefully monitor the patient when given systemic treatment. In addition, it offers benefits like ease of application, lower adverse reaction incidence, and an attractive cost-benefit ratio (38, 42). Despite the development of topical paromomycin in the 1980s, there have been few advances in the treatment of CL (23). BPQ has suitable physicochemical properties for topical delivery, such as low molecular weight and melting point (23). The excipients used in the formulation were inspired by the study by Mesquita et al. (43), which showed no toxic effects in mice (43).

The *in vivo* study showed that the combination therapy (topical nonionic cream containing BPQ 0.1% and 4-NB 0.1%) was effective against *Leishmania (Leishmania) amazonensis*, reducing the parasite burden of the lesions by 57% (bioluminescence analysis) compared to the untreated control. In this study, five groups were established: control (untreated), treated with the standard drug Glucantime, vehicle control (formulation without active ingredient), BPQ control (formulation BPQ only), and 4-NB control (formulation 4-NB only). The Glucantime group achieved a significant 95% reduction in lesion parasite burden compared to the control, but the intralesional method is invasive and painful. All other groups, including the combination, showed no statistical difference compared to the control group (untreated). It was observed that some groups, such as those treated with vehicle control or buparvaquone alone, showed elevated standard deviations, which compromised the statistical analysis across all study groups. This variability can be explained by the limitations of the *in vivo* model used (BALB/c mice infected with *L. amazonensis*), as this is an extremely susceptible animal model with an insufficient follow-up period for complete lesion healing (44). Despite the lower efficacy of the combination treatment compared to Glucantime, the avoidance of parenteral antimonials would greatly increase patient compliance and reduce treatment costs (23). Thus, the introduction of a topical formulation would be a significant advance for the treatment of simple CL (23).

In conclusion, this study demonstrated for the first time the *in vitro* activity of 4-NB against promastigote forms of *Leishmania (Leishmania) amazonensis*, evidenced by the reduction of UQ. Furthermore, the combination of 4-NB with BPQ demonstrated a significant potentiation of the activity against amastigote forms. The most promising result was observed *in vivo* studies, where combination therapy reduced 57% of the parasite burden in the lesions. These findings open possibilities for future studies to develop therapeutic alternatives and highlight the importance of investigating drug combinations in neglected diseases.

## MATERIALS AND METHODS

### Drugs and chemicals

Roswell Park Memorial Institute medium (RPMI 1640), fetal bovine serum (FBS), and HEPES were purchased from Gibco by Life Technologies (Brazil), and Schneider’s Drosophila medium was purchased from Lonza (Brazil). BPQ was kindly provided by Professor Dr. André Gustavo Tempone Cardoso (Instituto Butantan, Brazil), and Glucantime Sanofi-Aventis (Brazil) was kindly provided by Professor Dr. Silvia Reni Bortolin Uliana (University of São Paulo, Brazil). CM-H_2_DCFDA was purchased from Invitrogen Thermofisher (USA). Petroleum ether, hexane, and other reagents were purchased from Merck (Brazil). One-Glo luciferase assay substrate and Vivo-Glo *in vivo* grade luciferin were purchased from Promega (USA). The topical formulations were prepared at Ponto de Apoio Pharmacy (São Paulo, Brazil). UQ 6 and UQ 8 were purchased from Avanti Polar Lipids (USA). UQ 7 and UQ 9 were purchased from Sigma-Aldrich (USA). 4-NB, amphotericin B, miltefosine, phorbol-12-myristate-13-acetate, 3-[4,5-dimethylthiazole bromide]-2-yl]−2,5-diphenyl tetrazolium (MTT), dimethyl sulfoxide (DMSO), and other analytical reagents were purchased from Sigma-Aldrich (Brazil).

### Experimental animals

Female BALB/c mice were obtained from the Animal Facility of the University of São Paulo (Brazil). Animals were maintained in sterilized cages under a controlled environment and received water and food *ad libitum*.

### Parasites and cell lineage

The strain used was wild-type *Leishmania (Leishmania) amazonensis* (MHOM/ BR/1973/M2269), isolated from a patient in 1973 (state of Pará, Brazil). Promastigote forms were grown in M-199 medium supplemented with 10% FBS and 0.25% hemin at 25°C. The promastigote forms of the *Leishmania (Leishmania) amazonensis* transgenic line expressing luciferase (La-LUC) were grown in the same medium supplemented with 32 µg/mL hygromycin B under the same conditions, and the maximum passage number used was 10 (43). The human monocytic cell line THP-1 ATCC TIB-202 was cultured in RPMI medium with 20% inactivated FBS and 2 mM glutamine, maintained in a humid incubator at 37°C with 5% CO_2_ saturation. The maintenance of this cell line was performed three times a week, not allowing the culture to reach 1 × 10^6^ cells/mL, and the maximum passage number used was 20.

### *In vitro* anti-*Leishmania* activity, mammalian cytotoxicity, and selectivity index of BPQ and 4-NB

To determine the EC_50_ against *Leishmania (Leishmania) amazonensis* and the CC_50_, BPQ was solubilized in DMSO, amphotericin B and miltefosine were solubilized in sterile water, 4-NB was solubilized in medium (acidified and alkalized to solubilize), pH 7.2, and sterilized.

#### Promastigotes

Drugs were diluted with medium in 96-well microplates at the highest concentration of BPQ (0.2 µM), amphotericin B (1 µM), miltefosine (200 µM), and 4-NB (8 mM) in serial twofold dilutions. Promastigote forms in the late growth phase (non-stationary) were washed in Schneider medium and seeded at 2 × 10^6^/well. Miltefosine and amphotericin B were used as standard drugs. The microplate was incubated for 72 hours at 25°C. The viability of promastigote forms was evaluated by MTT assay (45) by reading the optical density in a plate reader (POLARstar Omega, BMG Labtech, Ortenberg, Germany) at 595 and 690 nm (46).

#### Intracellular amastigotes

Drugs were diluted in RPMI medium with 20% inactivated FBS and 2 mM glutamine using 96-well microplates at the highest concentration of BPQ (20 µM), amphotericin B (4 µM), miltefosine (200 µM), and 4-NB (4 mM) in serial twofold dilutions. THP-1 monocytes were induced to differentiate into macrophage-like cells using PMA. The cells were seeded at 5 × 10^4^ cells/well in 96-well plates (NUNC) in RPMI medium with 80 nM PMA, 20% inactivated FBS, 2 mM glutamine, and 10% THP-1 culture supernatant and incubated for 72 hours at 37°C with 5% CO_2_. The monocytes that had differentiated into macrophage-like cells were then infected with promastigote forms of La-LUC in the stationary phase at a ratio of 20:1 promastigotes/macrophage in RPMI medium with 20% inactivated FBS and 2 mM glutamine for 24 hours at 33°C with 5% CO_2_. The non-internalized parasites were removed, and the infected cells were treated with the drugs described above for 72 hours under the same conditions, plus 20 nM PMA. To quantify parasite load, we added the substrate luciferin, using the One-Glo luciferase assay in 96-well microplates (47). Luminescence units were determined using the same plate reader. The luminescence reading of uninfected macrophages was used as a baseline. Untreated infected macrophages and infected macrophages treated with the standard drugs were used as controls.

#### Cytotoxicity

The drugs were diluted in RPMI medium in 96-well microplates at the highest concentration of BPQ, amphotericin B, and miltefosine (200 µM) and 4-NB (20 mM) at twofold serial dilutions. THP-1 monocytes were differentiated into macrophage-like cells with PMA as described. Drug treatment was performed for 72 hours at 37°C with 5% CO_2_. The viability of macrophage-like cells was evaluated with the MTT assay, the same form described, and CC_50_ was calculated. To determine the selectivity of drugs, the SI was calculated using the following ratio: CC_50_ against human cell/CE_50_ against intracellular amastigotes.

### *In vitro* combination therapy with BPQ and 4-NB against *Leishmania (Leishmania) amazonensis*

#### Promastigotes

The interaction between BPQ and 4-NB in promastigote forms was evaluated using the modified isobologram method (19, 48). The EC_50_ values of two drugs alone were determined at 72 hours, and these values determined the maximum concentrations of individual drugs (19). The proportions used were 5:0, 4:1, 3:2, 2:3, 1:4, and 0:5 of BPQ and 4-NB, as described by Mesquita et al. (43). To determine the nature of the interaction and construct the isobologram, the fractional inhibition concentration index was calculated by the ratio between the EC_50_ values of the combined and the EC_50_ values of the drug alone. Isobolograms were constructed by plotting the FIC for each drug ratio. The sum of the FIC (∑FIC) and the mean sum of FIC (X∑FIC) were calculated. Interactions were classified as synergistic (X∑FIC ≤ 0.5), additive (X∑FIC between >0.5 and ≤4), or antagonistic (X∑FIC > 4) as recommended by Odds (26).

#### Intracellular amastigotes

The potentiation between BPQ and 4-NB in amastigote forms was investigated by a method different from the previous one. Only BPQ showed activity of macrophage-like cells infected with intracellular amastigote forms after 72 hours. As 4-NB did not show activity in amastigote forms under the conditions tested, a drug potentiation study was performed using fixed doses of 4-NB (2, 1, 0.5, and 0.25 mM). This study was designed to verify that the EC_50_ value of BPQ can be reduced in the presence of 4-NB. The potentiation was calculated from the ratio of the EC_50_ values of the combined and the EC_50_ values of the drug alone.

### *In vitro* study of the mechanism of action of 4-NB against *Leishmania (Leishmania) amazonensis*

#### Metabolic labeling

UQs were identified using two metabolic labels: labeling with 0.5 µCi/mol of radioactive 4-hydroxybenzoic acid [ring-^14^C] (American Radiolabeled Chemicals, Inc., ARC1570, Royston, Herts, UK) and 2.0 µCi/mol of the radioactive precursor mevalonic acid, ammonium salt R-[5-^3^H] in promastigote cultures. For this purpose, 2 × 10^9^ promastigote forms were treated with two concentrations, one concentration equivalent to the EC_50_ value, and another with half of the concentration equivalent to the EC_50_ value (sublethal dose, which is represented as EC_25_) for 72 hours (total time), during which the culture medium and drugs were changed every 24 hours. After 48 hours of treatment, the promastigote forms were washed, incubated with drugs, and labeled with respective radioactive labels for 24 hours (totaling 72 hours), after which the promastigote forms were washed and frozen at −80°C (only pellet). Parasites were disrupted by freezing in liquid nitrogen and thawing at 37°C, and the UQ extraction was carried out under agitation using water (50 µL), petroleum ether (400 µL), and methanol (600 µL). After centrifugation at 1,000 *g* for 3 minutes at 4°C, the upper phase was collected in a glass tube and dried in a nitrogen atmosphere at room temperature. The extracted material was resuspended in methanol and analyzed by RP-HPLC. Standard UQ homologs (UQ 6, 7, 8, and 9) were co-injected with the samples of extracts. RP-HPLC (Gilson) was used, and in all RP-HPLC methods, the fractions were separated using an FC203B collector (Gilson). The same amounts of treated and untreated metabolically labeled promastigotes were applied to the column. The software used for data processing was Trilution LC System Software (Gilson). The compounds were detected by absorbance using a Gilson 170 diode array detector (DAD) (Gilson, Villiers-le-Bel, France) operating at different absorbances: 200, 210, 230, 250, 265, 275, 290, and 340. The mobile phase used was methanol:hexane (9:1, vol/vol) at a flow rate of 1.2 mL per minute, and the stationary phase was a 250 mm × 4.6 mm × 5 µm Phenomenex Luna C18 column (Phenomenex, CA, USA) as described by Zafra et al. (49). After collecting the samples/fractions, they were dried in an oven at 50°C, suspended in 500 µL of liquid scintillation mixture (Perkin Elmer Life Sciences, MA, USA), and analyzed using a Tri-Carb 5110TR Liquid Scintillation Counter (Perkin Elmer Life Sciences, MA, USA). This protocol was based on and adapted from Verdaguer et al. (25), Zafra et al. (49), and Sussmann et al. (50).

#### Identification of UQ

To identify which ubiquinones are present in the promastigote samples, a LC/MS/MS technique was applied using TSQ Quantum MAX Triple Quadrupole mass spectrometer coupled with an Acella 600 UHPLC system (Thermo Fisher Scientific, Waltham, MA, USA). Positive electrospray ionization was performed using the commercial standards of UQ 6, UQ 7, UQ 8, and UQ 9. Promastigote extracts from 6 × 10^10^ parasites were prepared, as described previously. A mobile phase of 100% methanol containing ammonium formate (5 mM) was used for isocratic separation of the analytes, performed on a Phenomenex Luna C18 column (5 µm, 250 mm × 4.6 mm) at a flow rate of 1 mL·minute^−1^ at room temperature. Multiple reaction monitoring (MRM) technique was applied, which involves the ionization of the sample, the selection of precursor ions at specific *m/z* (mass-to-charge ratio), fragmentation, and the analysis of product ions. The precursor and product ions selected and used for the identification of the commercial standards were 591 *m/z* > 197 and 154 *m/z* for UQ6; 659 *m/z* > 197 and 151 *m/z* for UQ7; 727 *m/z* > 197 and 154 *m/z* for UQ8; and 795 *m/z* > 197 and 237 *m/z* for UQ9. Based on the study by Ruiz-Jiménez et al. (51), the 197 *m/z* production is common for all ubiquinones and could be monitored in the total ion current (TIC) mode for all analyses (51). For the LC-MS/MS method, the mass spectrometer operational parameters were set as follows: spray voltage, 3,000 V; sheath and auxiliary gas pressure, 30 and 5 arbitrary units, respectively; ion transfer capillary temperature, 350°C; vaporizer temperature, 350°C; collision gas pressure, 1.5 mTorr; source CID, 4 V; and Q1 and Q3 peak width (FWHM): 0.7 Da. These settings were optimized for maximum sensitivity and selectivity for the compounds of interest. The raw data were analyzed by XCalibur (Thermo Fisher).

#### Mitochondrial membrane potential

Promastigote forms were incubated (2 × 10^7^/well) with 4-NB at the EC_50_ value for various times (1, 24, 48, and 72 hours) at 25°C. After this, the parasites were labeled with JC-1 at a concentration of 10 µg/mL for 10 minutes at 37°C. The parasites were then washed and read using the same plate reader, with an excitation filter of 485 nm and emission filters of 530 and 590 nm, where mitochondrial depolarization is indicated by a decrease in the ratio of 590 to 530 nm. Untreated parasites were used as a control, and parasites treated with FCCP at 20 µM for 1 hour were used as a control for mitochondrial membrane potential depolarization (52, 53). The tests were carried out in triplicate, and at least three different experiments were performed, calculating the mean and standard deviation between the tests.

#### Reactive oxygen species

Promastigote forms were incubated (2 × 10^7^/well) with 4-NB at the EC_50_ value for various times (1, 24, 48, and 72 hours) at 25°C. At the end, the parasites were labeled with CM-H_2_DCF-DA probe (5 µM) and incubated for 15 minutes, washed, and read using the same plate reader, with an excitation filter of 485 nm and emission of 520 nm (43). Untreated parasites were used as a control, and as positive control for increased ROS production, parasites were treated with sodium azide (10 mM) at 1 hour (54). The tests were carried out in triplicate, and at least three separate experiments were carried out, calculating the mean and standard deviation between the tests.

#### Ultrastructural evaluation

To assess the damage caused by 4-NB, promastigote forms (nonstationary) at 4 × 10^7^/well were incubated with 4-NB at its respective EC_50_ value for 1, 24, 48, and 72 hours at 25°C. The parasites were fixed in a 2.5% glutaraldehyde in a 0.1 M sodium cacodylate buffer at pH 7.2, post-fixed in 1% osmium tetroxide, gradually dehydrated in acetone, and embedded in Epon 812 resin (55). Ultrathin sections were stained with 2% uranil acetate and lead citrate. The material was examined in a transmission electron microscope JEM 1011 (Jeol, Tokyo, Japan) at 80 kV, and images were recorded with a Gatan 785 ES1000W Erlangshen camera. The untreated parasites were used as a control, and as 4-NB was solubilized in the culture medium, no solvent control was used.

### *In vivo* activities of the combination of BPQ with 4-NB against *Leishmania (Leishmania) amazonensis*

To evaluate the efficacy of the combined drugs *in vivo*, the topical route of administration was chosen. Female BALB/c mice (6 week-old) were inoculated with 1 × 10^8^ La-LUC promastigote forms (stationary phase) at the base of the tail. Five weeks after infection, when the lesions had already been established, the lesions were measured, the animals were homogeneously distributed in groups, and the treatment was started (43, 56). The animals were inoculated at the base of the tail to facilitate the administration of the topical formulation and to prevent removal of the formulation for the animals (43). The animals were individually housed in boxes containing sterile absorbent paper, and the paper was changed daily. The topical formulations were prepared at the Ponto de Apoio Pharmacy (São Paulo, Brazil), containing 4% propylene glycol, 4% medium-chain triglycerides (caprylic acid and capric acid), 8% distilled water, 84% non-ionic cream (Nostrabase, Infinity pharma), 0.1% BPQ, and 0.1% 4-NB. Another three topical formulations were prepared for experimental control. The animals were treated with approximately 120 mg of the topical formulation once a day for 28 consecutive days, except for the group treated with Glucantime (14 days of treatment). Six distinct groups were used in this experiment, with five mice per group. The following groups were used: A—control group (untreated); B—group treated with the standard clinical drug Glucantime intralesional (treated 50 mg/kg of body weight subcutaneously for 14 days) (57); C—vehicle control group (topical formulation with no active ingredient); D—BPQ 0.1% drug control group; E—4-NB inhibitor 0.1%; and F—test group (combination of BPQ 0.1% with 4-NB 0.1%). Disease progression was assessed every 7 days by measuring lesion size (mean lesion width and height) in millimeters using a micrometer (Mitutoyo Corporation, Japan), and the body weight of each animal was also assessed weekly (internal controls of the experiment). The efficacy of the treatment was evaluated at the end of treatment by luciferase detection using a bioimaging system (IVIS Spectrum; Caliper Life Sciences, Inc., MA, USA) as described previously by Reimão et al. (47). At the end of the experiments, the animals were euthanized by a chemical method approved by the CEUA.

### Statistical analyses

The EC_50_ was determined by sigmoidal regression curves using GraphPad Prism 5.0 software. Differences between samples were compared with controls and statistically evaluated using the one-way ANOVA (Tukey’s multiple comparison test) to determine the *P*-value. The *P*-value of <0.05 was considered significant.
